# Pain Distraction During Awake Major Colorectal Surgery: Supporting Patients Beyond the COVID-19 Era. Preliminary Findings

**DOI:** 10.3389/fsurg.2021.754059

**Published:** 2021-09-17

**Authors:** Andrea Romanzi, Gaetano Gallo, Sabrina De Rango, Barbara Vignati, Alberto Vannelli

**Affiliations:** ^1^Department of General Surgery, Valduce Hospital, Como, Italy; ^2^Department of Medical and Surgical Sciences, University of Catanzaro, Catanzaro, Italy; ^3^Department of Anesthesiology and Critical Care, Valduce Hospital, Como, Italy; ^4^Department of Clinical and Biomedical Sciences “Luigi Sacco”, University of Milan, Milano, Italy

**Keywords:** awake surgery, pain distraction, loco-regional anaesthesia, combined spinal-epidural anaesthesia, mobile theatres, colorectal surgery, case report, COVID-19

## Abstract

**Introduction:** During the coronavirus disease 2019 (COVID-19) pandemic, hospitals rapidly ran out of intensive care beds. Because minimally invasive surgery and general anaesthesia are both aerosol-generating procedures, their use has become controversial. We report a case series of awake undelayable colorectal surgeries which, innovatively, took advantage of intraoperative pain distraction. Moreover, we describe our frugal solution to social distancing in psychological support of inpatients.

**Methods:** Between October 2020 and February 2021, five patients underwent acute-care colorectal surgery under locoregional anaesthesia in our department. A 3D mobile theatre (3DMT) was used during the operation to distract the patients from pain. Vital signs, pain intensity, ergonomic comfort/discomfort, sense of presence and distress were intraoperatively monitored. A postoperative “cuddle delivery” service was instituted: video messages from relatives and close friends were delivered daily to the patient through the 3DMT. Emotional effects were investigated through clinical interviews conducted by a psychologist at our hospital.

**Results:** Both intraoperative and postoperative pain were always well controlled. Conversion to general anaesthesia and postoperative intensive support/monitoring were never necessary. The “cuddle delivery” initiative helped patients fill the emotional gap created by the strict containment measures implemented inside the hospital, distracting them from emotional anxiety and physical pain.

**Conclusions:** During the next phase of the COVID-19 pandemic and even after the COVID-19 era, awake laparotomy under locoregional anaesthesia may be a crucial option for delivering acute-care surgery to selected patients when intensive care beds are unavailable and postponing surgery is unacceptable. We also introduce a new modality for the provision of emotional support during postoperative inpatient care as a countermeasure to the restrictions imposed by social distancing measures.

## Introduction

During the coronavirus disease 2019 (COVID-19) pandemic, allocating intensive care beds to patients needing acute-care surgery became very difficult. After the first lockdown, innovative COVID-19 preoperative triage protocols allowed a gradual reopening and the ramping up of elective surgeries (1).

Major abdominal surgeries are generally carried out with minimally invasive surgery (MIS) under general anaesthesia (GA). MIS and GA are both aerosol-generating medical procedures (AGMPs), and their use has become controversial during the pandemic because they could contribute to the spread of pathogens inside operating theatres (2, 3). In addition, frail patients may require intensive postoperative monitoring/support, which cannot be provided when resources are scarce (4). In such a unique context, performing open abdominal surgery under locoregional anaesthesia (LA) helped us perform acute-care surgery in selected patients during the COVID-19 pandemic.

LA (spinal, epidural or combined spinal-epidural) reduces the exposure of medical staff to patients' respiratory secretions and the risk of perioperative viral transmission and preserves patients' cardiorespiratory function.

Besides this, the implementation of containment measures and social distancing resulted in serious consequences for our inpatients: the impossibility of being visited by their loved ones (sometimes for longer than a week) clearly increased the sense of solitude, discouragement, and depression in almost all our inpatients, especially the elderly patients. This negatively influenced their postoperative course.

The aim of this study was to investigate the possibility of distracting patients from pain through the use of a 3D mobile theatre (3DMT) as a means of improving the approach to performing awake major abdominal surgeries. Moreover, because we believe emotional care is fundamental (especially for the elderly population), we report the use of a functional countermeasure to the effects of social distancing to support the emotional needs of inpatients.

## Methods and Results

Five patients needing acute-care surgery for colorectal disease were treated at our Department between October 2020 and February 2021 (Table 1).

**Table 1:** Patients' clinico-pathological characteristics and intraoperative results.

**Pt (#)**	**Age**	**Sex**	**Diagnosis**	**ASA score**	**Surgery**	**OT (min)**	**S**	**CGA**	**3DMT (min)**	**PO ICU**
1	64	♂	RCa (nCRT)	II	LAR (sanCRT)	170	–	–	90	–
2	84	♀	CD	III	SR	85	yes	–	60	–
3	76	♂	CCa	III	RC	90	yes	–	60	–
4	67	♂	CCa	III	RC	105	–	–	60	–
5	92	♀	RCCa	III	RC	75	–	–	60	–

One week prior to each surgery, on the day on which the preadmission tests were performed, both the surgical procedure and approach to anaesthesia were explained to the patients during a multidisciplinary meeting. On that occasion, each patient underwent nasopharyngeal swab sampling to test for COVID-19 (all were negative), and the 3DMT was shown to the patient. Each patient had the opportunity to wear the device, learn how to adjust it, and express his/her approval of its use during surgery after being fully informed of the risks and benefits.

### Awake Laparotomy

Surgery was performed under combined spinal-epidural (CSE) anaesthesia. Continuous epidural analgesia was administered with an elastomeric pump. The protocols for the administration of CSE anaesthesia and the elastomeric pump settings adhered to the routine clinical practice in our institution (5, 6).

Vital signs, intraoperative pain intensity, ergonomic comfort/discomfort level, sense of presence and distress were continuously monitored. Only light sedation was administered (midazolam 5 mg) to two patients. No other drugs were administered to the patients during surgery. Intraoperative pain was always well-controlled (VAS ≤ 3), and conversion to GA was unnecessary.

Postoperative pain was assessed daily, and pain control was satisfactory (VAS ≤ 3). Intensive postoperative monitoring/support was never necessary. A separate COVID-19-free ward was established for postoperative recovery to ensure that COVID-negative patients remained isolated from all other patients. The epidural elastomeric pump was removed on postoperative day (POD) 3 for all patients. Patients were discharged free from complications on POD 5 (mean value).

### 3D Mobile Theatre

During surgery, patients wore Royole's Moon (RM) (Royole®, Shenzhen, China). RM is an all-in-one 3DMT headset. It uses two AMOLED displays that deliver 3D or 2D content in full HD 1080p resolution. The optics are independent and can be adjusted from −7.0 D to +2.0 D. An immersion mask is mounted on the device to ensure a close fit around the eyes. Active noise cancellation was used. The right ear pad contains flexible sensor technology that can be used to navigate the menu and adjust the volume by swiping or tapping a finger on it.

Internal flash storage allowed the storage of several 4K ultra HD videos. Some videos offered immersion in a natural setting from an aerial perspective, and other videos simulated walking through a specific scenario.

**Figure 1:**
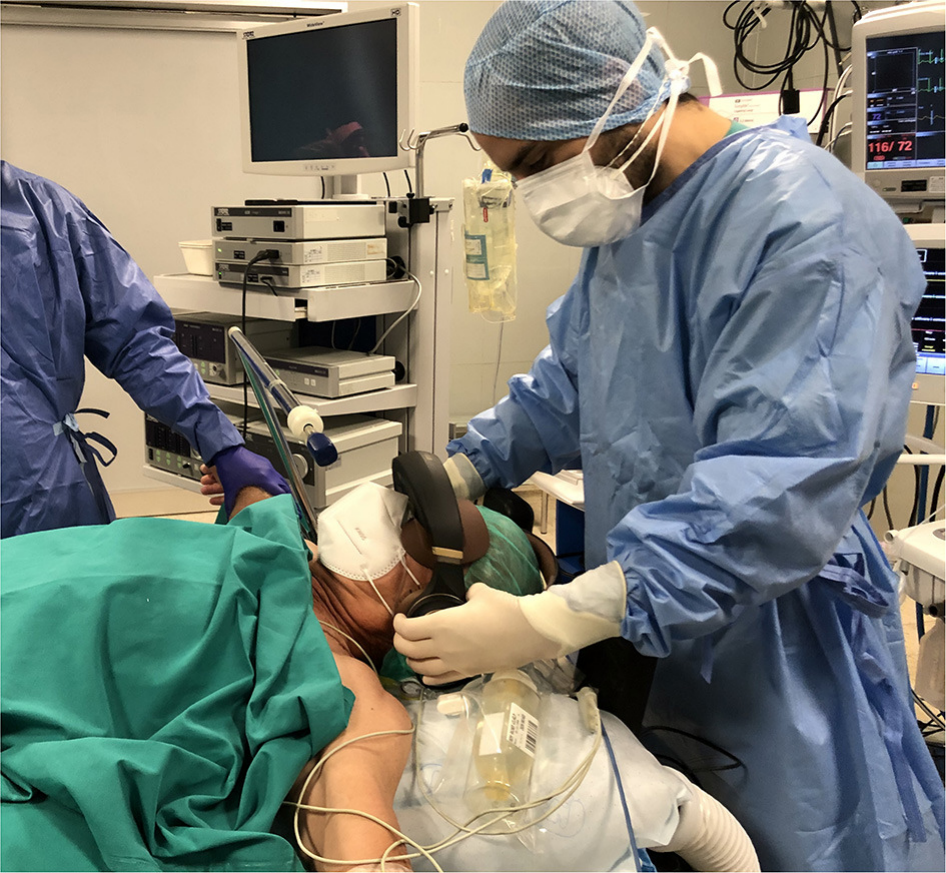
Patient wearing the device (preoperative snapshot).

**Figure 2:**
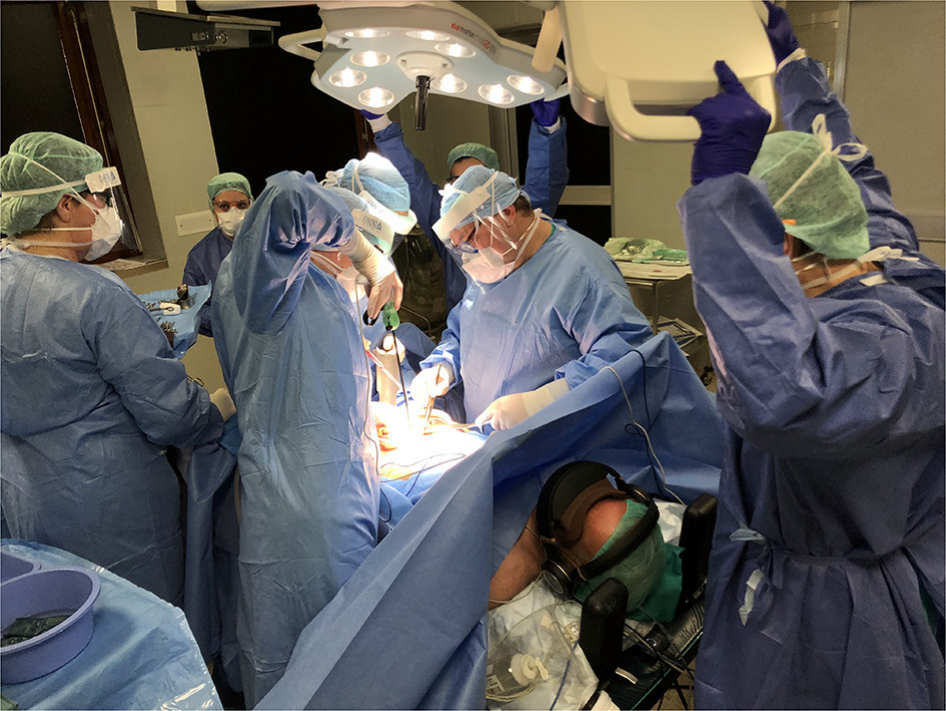
Intraoperative use of the device.

After the patient was positioned on the operating table, the patient's dominant hand was freed to enable the patient to adjust the device in case of displacement. Patients wore the 3DMT two times during surgery (Figures 1, 2). The first time started before the surgical incision was made and lasted 40 min. After a pause of 20 min, they wore the 3DMT again for 20 min. Patient #1 wore the 3DMT longer because he underwent a prolonged surgery: the second time, he wore the 3DMT for 50 min.

A questionnaire was designed to investigate critical factors that may have affected patients during or after the use of the device. The questionnaire was completed by each patient before discharge. Questionnaires were then analysed (Table 2).

**Table 2:** Sample of the evaluation sheet showing the mean mark for each aspect.

		**1–4**	**5–6**	**7–10**
**First impression**	1. lightness of the device	■	□	□
	2. comfort	□	□	■
	3. ease to focus	■	□	□
	4. video quality	□	□	■
	5. audio quality	□	□	■
	6. ease to wear the device	□	□	■
	7. ease to use the device (in general)	□	■	□
**While watching**	8. sense of constriction	■	□	□
	9. discomfort (□ nasal ■ ocular □ auricular)	□	■	□
	10. pain (□ nasal □ ocular □ auricular)	■	□	□
	11. steadiness of the device on the face	□	□	■
**At removal**	12. memory of the weight of the device	■	□	□
	13. discomfort (□ nasal □ ocular □ auricular)	■	□	□
	14. pain (□ nasal □ ocular □ auricular)	■	□	□
	15. eye strain	■	□	□
	16. eye dryness	■	□	□
	17. headache	■	□	□
	18. dizziness	■	□	□
	19. nausea	■	□	□
	20. tinnitus	■	□	□

*Patients expressed their personal evaluation from 1 (completely negative evaluation or absence of the sensation in question) to 10 (completely positive evaluation or presence of the sensation in question). Scores were interpreted inversely based on the positive or negative nature of the factor in question: scores from 1 to 4 were considered indicators of a negative impression if the question pertained to a positive factor (or a positive impression in the case of a negative factor); scores of 5 and 6 were considered indicators of a neutral impression; and scores from 7 to 10 were considered indicators of a positive impression if the question pertained to a positive factor (or a negative impression in the case of a negative factor)*.

### Cuddle Delivery Initiative

Before admission, we contacted the relatives and close friends of each patient and gave them the opportunity to send us homemade videos addressed to their loved one with the aim of cheering the patient. The video messages were delivered daily to the patients through RM during their postoperative stay. The day before discharge, patients underwent clinical interviews with the psychologist in our department.

## Discussion

Multiple authors have described awake laparotomy as a feasible and safe approach to major surgical procedures; hence, this solution has been considered a valid option when gradually making surgery available again after its temporary cessation during the COVID-19 pandemic (2, 5–7). Some colleagues even reported that awake laparoscopy is adequate and safe for minor laparoscopic surgeries in healthy patients (8). Nevertheless, the recent identification of SARS-CoV-2 in the peritoneal fluid of COVID-19 patients likely makes this impossible at the current time (9).

The use of head-mounted displays or portable virtual reality (VR) devices in medicine, surgery and behavioural healthcare is not new (10). In all cases, head-mounted displays without earphones were used. The effective use of these displays to distract patients from pain during prolonged and invasive surgeries has thus far only been hypothetical (10).

To the best of our knowledge, this is the first case series focusing on distraction from pain through the use of an immersive audio-visual device during awake major colorectal surgeries.

Several 3DMT units are commercially available. We selected this specific device because of some specific characteristics. First, RM is an audio-visual all-in-one headset. If immediate anaesthesiological support is required during surgery, the device can be quickly removed. Second, other popular head-mounted devices come with an elastic band that goes around the head. This can be a source of discomfort during prolonged supine positioning. Third, independent optics allow patients to view it without prescription glasses even if they have mild optical defects that differ between their eyes.

Intraoperatively, the large, curved, full-HD screen helped deliver videos with compelling stereoscopic depth perception. The noise-cancelling headphones together with the immersion mask blocked out the ambient sources of distraction and created an immersive experience. The optimised viewing angle and the combination of ultra-high resolution pictures with a fast image response rate contributed to providing relaxation during prolonged viewing by reducing eye strain. The ergonomic design ensured a comfortable fit: the patients never complained of a sense of constriction or breathing restriction. The analysis of the questionnaires revealed that, despite being initially discouraged by the weight of the device and hesitant during the first attempt to focus the device, after proper training, patients did not encounter any difficulties in its use or discomfort during or after its use (Table 2).

Additionally, on the basis of our experience, the use of 3DMT as a countermeasure to the negative effects of social distancing on patients appears to be a promising approach and represents an example of the positive application of technology (11). It is well-known that older patients affected by dementia can develop postoperative confusion, disorientation, depression and fear (3). Social distancing made it impossible for patients to have visitors, exacerbating their feelings of solitude and discouragement. We have also noted psychological issues in the elderly patients without dementia and younger patients.

The psychological interviews revealed that every patient appreciated the initiative, reporting that it helped reduce their sense of loneliness and increased their desire to return home. The patients also reported that the video messages distracted them from physical pain. None of our patients showed signs of depression. None of our patients required the administration of benzodiazepines or other anti-anxiety medications during hospitalisation. These elements lead us to believe that postoperative emotional services may have marked positive effects on the postoperative course.

Our study has some limitations. It was a single-centre study based on a small group of patients. Nevertheless, we believe our preliminary data may allow to make valuable observations and to raise useful questions. The effects of emotional and psychological postoperative support were only investigated through a clinical interview; further studies including a tailored psychological questionnaire are needed to standardise the evaluation and objectively assess the impact of emotional support on the postoperative course.

During the pandemic, open surgery under LA is a crucial option for delivering acute-care surgery when ICU beds are unavailable and postponing surgery is unacceptable. After the COVID-19 era, methods of distracting patients from pain may make awake surgery (whether open or minimally invasive) more pleasant for the patient. Moreover, the use of a 3DMT can help deliver postoperative psychological care when social distancing measures are in place.

## Data Availability Statement

The original contributions presented in the study are included in the article/Supplementary Material, further inquiries can be directed to the corresponding author/s.

## Ethics Statement

The studies involving human participants were reviewed and approved by Valduce Hospital IRB. The patients/participants provided their written informed consent to participate in this study. Written informed consent was obtained from the individual(s) for the publication of any potentially identifiable images or data included in this article.

## Author Contributions

AR, AV, and SD designed the study and analysed and interpreted the patient data. AR, GG, and BV performed the review of the manuscript and were major contributors in writing the manuscript. All authors read and approved the final manuscript.

## Conflict of Interest

The authors declare that the research was conducted in the absence of any commercial or financial relationships that could be construed as a potential conflict of interest.

## Publisher's Note

All claims expressed in this article are solely those of the authors and do not necessarily represent those of their affiliated organizations, or those of the publisher, the editors and the reviewers. Any product that may be evaluated in this article, or claim that may be made by its manufacturer, is not guaranteed or endorsed by the publisher.
