# Supplementary figures and images for: Pain Distraction During Awake Major Colorectal Surgery: Supporting Patients Beyond the COVID-19 Era. Preliminary Findings
Source: Front Surg. 2021 Sep 17;8:754059. doi: 10.3389/fsurg.2021.754059 (PMC8484703; doi:10.3389/fsurg.2021.754059)

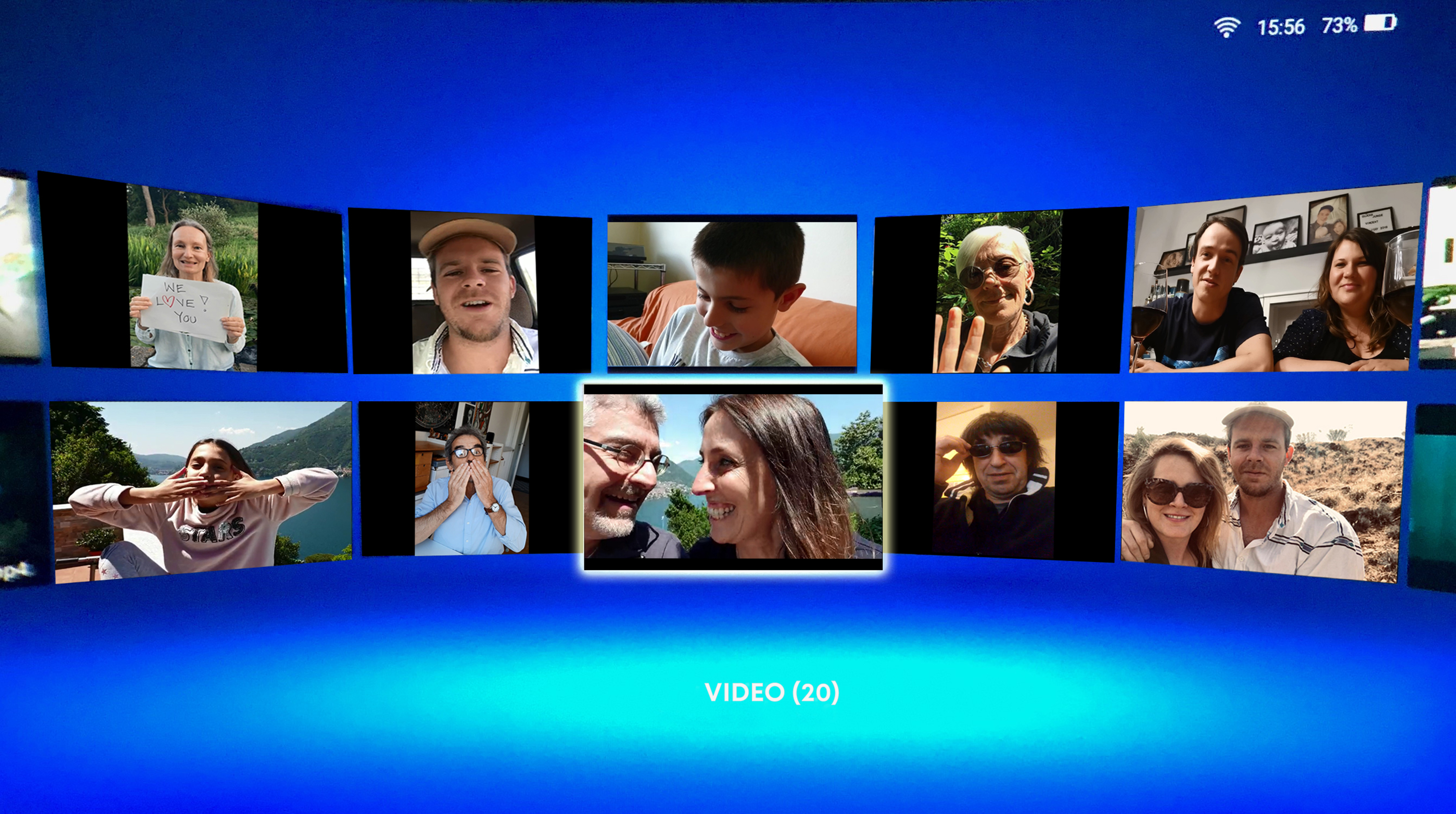

Supplement: Supplementary file 1 [file Image_1.jpg]
